# Supplementary material for: The non-canonical poly(A) polymerase FAM46C acts as an onco-suppressor in multiple myeloma
Source: Nat Commun. 2017 Sep 20;8:619. doi: 10.1038/s41467-017-00578-5 (PMC5606997; doi:10.1038/s41467-017-00578-5)
Supplement: Supplementary file 2 — Supplementary Information [file 41467_2017_578_MOESM2_ESM.pdf]

Description of Supplementary Files

File name: Supplementary Information
Description: Supplementary figures and supplementary tables.

File name: Supplementary Data 1
Description: Differential expression of annotated transcripts in list of transcripts that were differentially expressed in SKMM1 and H929 MM cell lines transduced with FAM46C^{WT}-GFP compared to FAM46C^{mut}-GFP transductions. Following columns with DESeq2 results are assigned to each comparison: 'baseMean' – average of the normalized count values, taken over mutant and wild type samples; 'log2FoldChange' – log2 fold change in the mutant comparing to wild type; 'lfcSE' – the standard error estimate for the log2 fold change estimate; 'stat' – Wald significance test statistics; 'pvalue' - significance test p-value; 'padj'– Benjamini-Hochberg adjusted p-value.

File name: Supplementary Data 2
Description: List of transcripts polyadenylated in SKMM1 and H929 MM cell lines transduced with FAM46C^{WT}-GFP compared to FAM46C^{mut}-GFP transductions. Columns represent "polyadenylation ratios" calculated by dividing the levels of individual mRNA in long poly(A) fractions (#5 and #6) relative to the short poly(A) fraction (#1) in wild-type FAM46C cells using relative levels observed in FAM46C^{mut} cells.

File name: Supplementary Data 3
Description: Mass spectrometry data from FAM46C immunoprecipitations.

File name: Supplementary Data 4
Description: List of oligonucleotides used in this study.

File name: Peer review file

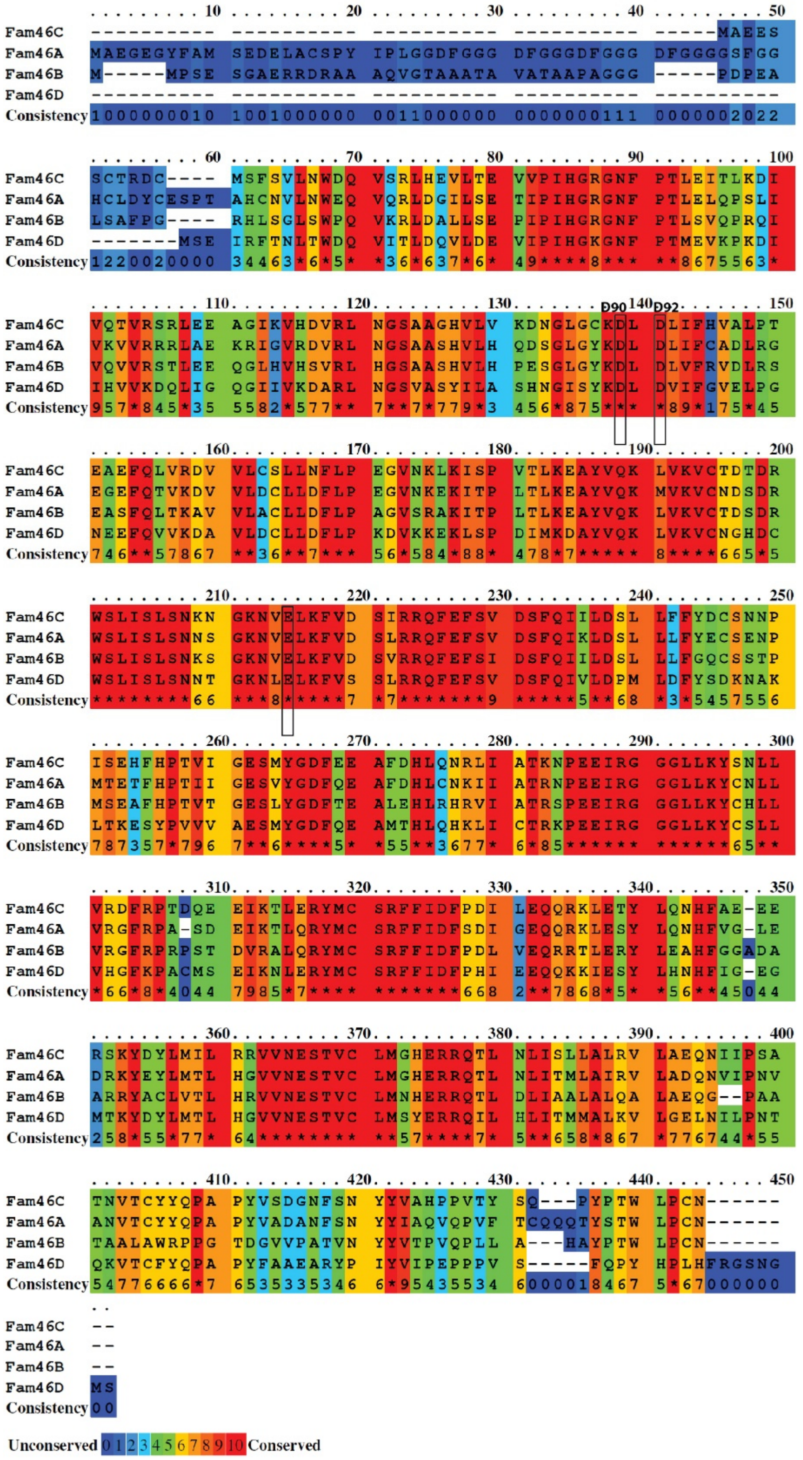

Supplementary Figure 1. Multiple sequence alignments color-coded for amino acid conservation of FAM46 protein family representatives in humans performed by PRALINE. The scoring color scheme starts at 0 for the least conserved alignment position up to 10 for the most conserved alignment position. Invariant catalytic residues from highly conserved NTase domain ([DE]h[DE]h are shown in boxes (D90 and D92 in case of FAM46C).

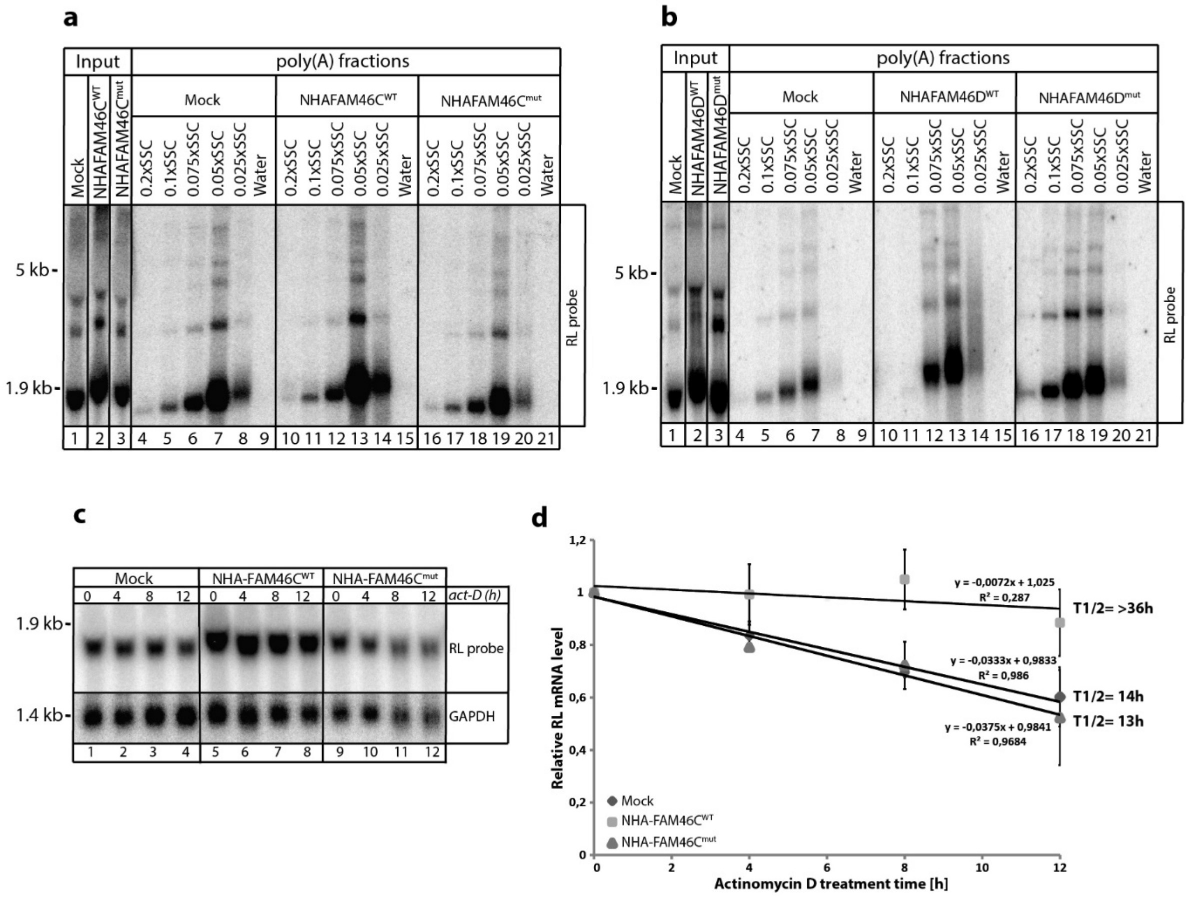

Supplementary Figure 2. (a) Northern blot analysis of RL mRNA from control HEK293 cells (lanes 4-9) after tethering of NHA-FAM46C^{WT} (lanes 10-15) or NHA-FAM46C^{mut} (lanes 16-21) which were fractionated based on the length of the poly(A) tails. Input RNAs are shown in lanes 1-3 (**b**) Northern blot analysis of RL mRNA from control HEK293 cells (lanes 4-9) after tethering of NHA-FAM46D^{WT} (lanes 10-15) or NHA-FAM46D^{mut} (lanes 16-21) which were fractionated based on the length of the poly(A) tails. Input RNAs are shown in lanes 1-3. (**c**) Time course of actinomycin-D treatment. Northern blot detection of RL transcript from: control HEK293 cells (lanes 1-4), after tethering of NHA-FAM46C^{WT} (lanes 5-8) and NHA-FAM46C^{mut} (lanes 9-12). GAPDH mRNA was used as loading controls for all northern blots. (**d**) RL mRNA half-life quantifications shown as a plot with SD error bars (n=3).

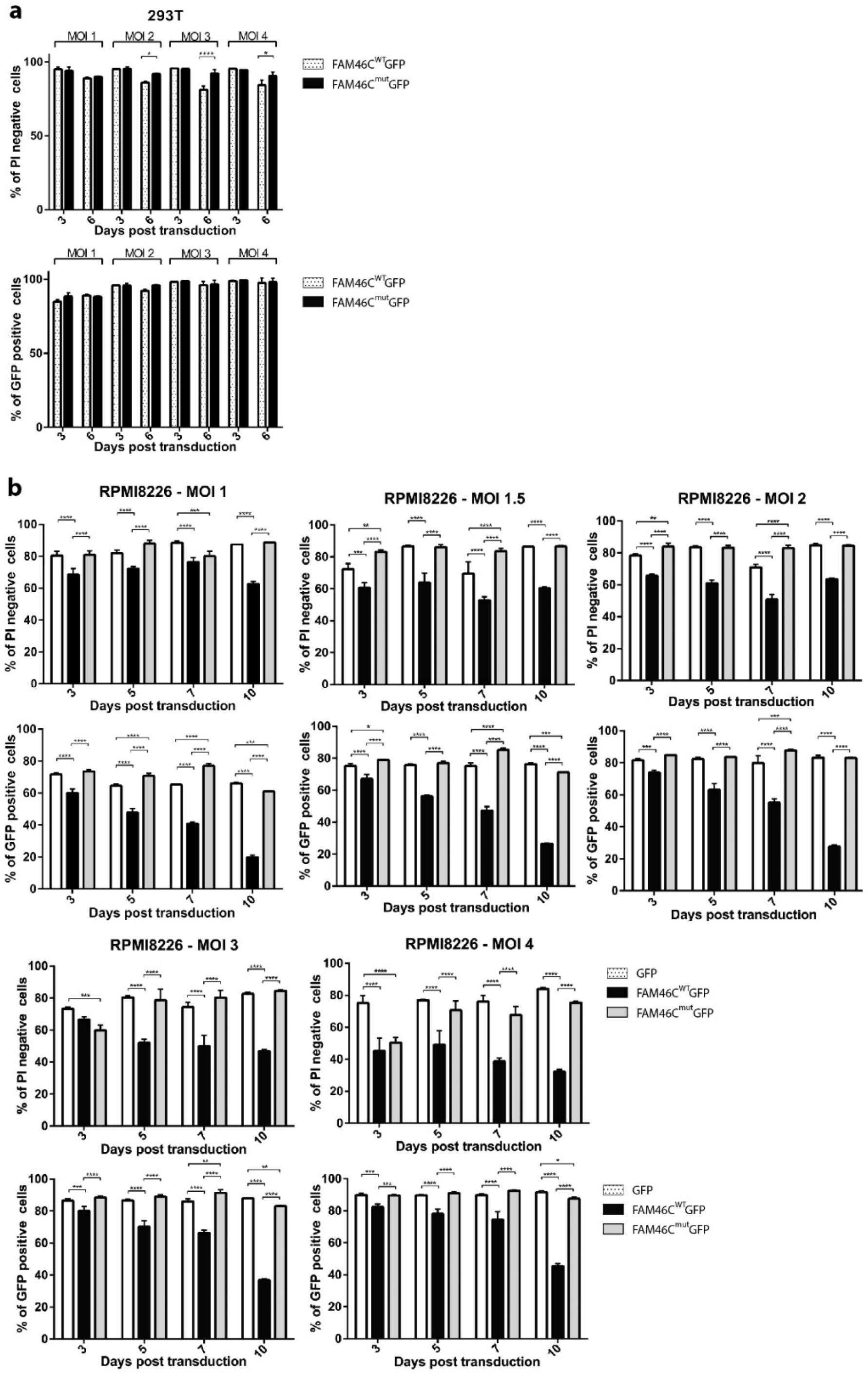

Supplementary Figure 3. Expression of FAM46C^{WT} induces multiple myeloma cell death. (**a, b**) Summary of flow cytometry analyses presented as bar graphs showing GFP expression levels and reduced viability of HEK293T (**a**) and RPMI8226 multiple myeloma cell line (**b**) depending on the amount of virus used for transduction and the time of expression analysis. Data are presented as percentage of cells ±SD (n=3). *P* values were calculated using 2-way ANOVA tests (**P*<0.05, ***P*<0.01, ****P*<0.001, *****P*<0.0001).

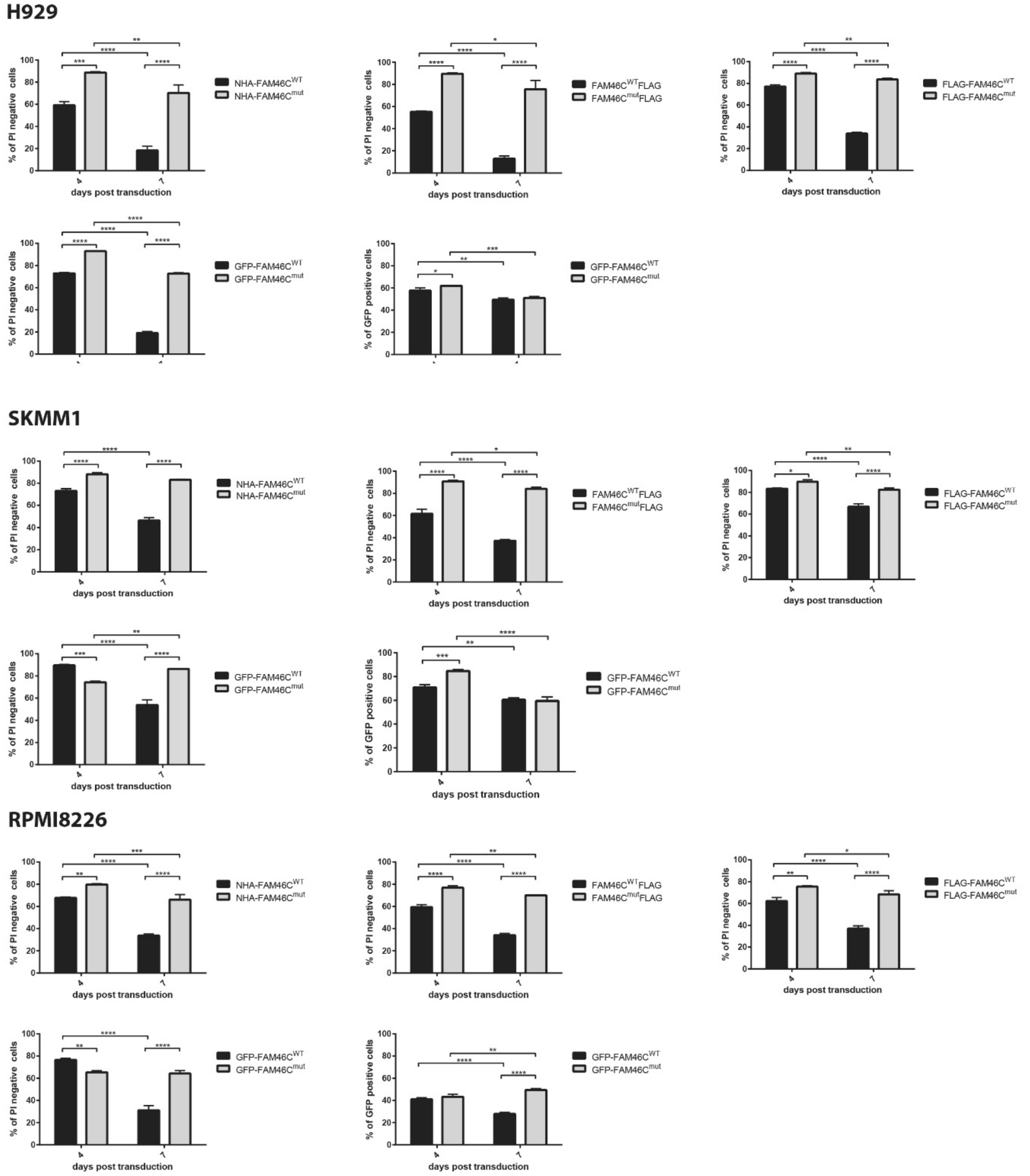

Supplementary Figure 4. Different tags at the N and C termini of FAM46C protein do not affect the function of the protein. Summary of flow cytometry analyses presented as bar graphs showing reduced viability of multiple myeloma cell lines (H929, SKMM1, and RPMI8226) throughout the time course of FLAG-FAM46C, FAM46C-FLAG, GFP-FAM46C and NHA-FAM46C expression. Data are presented as percentage of cells ±SD (n=3). *P* values were calculated using 2-way ANOVA tests (**P*<0.05, ***P*<0.01, ****P*<0.001, *****P*<0.0001).

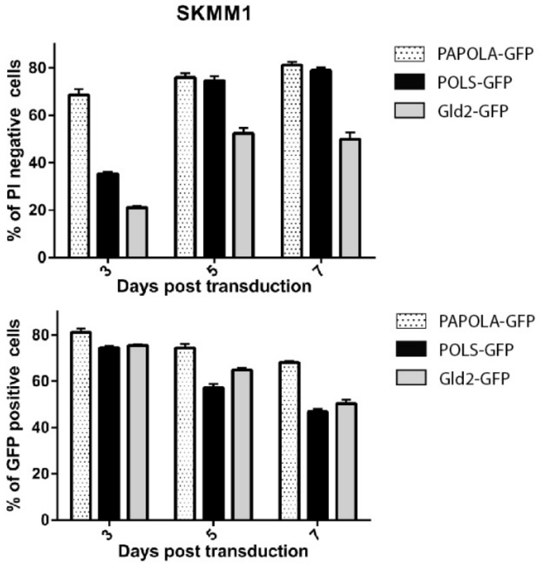

Supplementary Figure 5. Expression of PAPOLA-GFP, POLS-GFP, and GLD2-GFP constructs had no major effect on multiple myeloma cell death. Summary of FACS analyses presented as bar graphs showing GFP expression level and reduced viability of SKMM1 multiple myeloma cell lines transduced with a lentivirus carrying PAPOLA, POLS, or GLD2. Data are represented as percentage of cells ± SD (n=3).

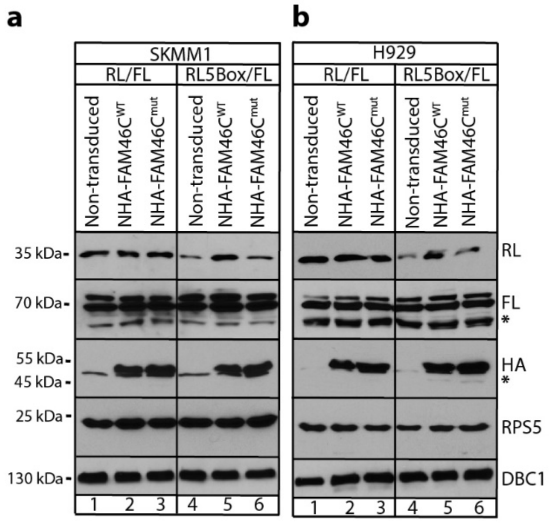

Supplementary Figure 6. FAM46C tethering enhanced expression of *Renilla* luciferase (RL) reporter in MM cells. SKMM1 **(a)** and H929 cells **(b)** expressing reporter genes of *Renilla* luciferase (RL) and Firefly luciferase (FL) or RL containing five boxB sites in its 3′-UTR (RL5Box) and FL were transduced with lentiviruses carrying $FAM46C^{WT}$ or $FAM46C^{mut}$ harboring the N-terminal λN boxB binding domain and HA-tag. Western blot detection of RL and FL proteins in non-transduced (**a, b**; lanes 1 and 4) and after NHA-$FAM46C^{WT}$ (**a, b**; lane 2 and 5) or NHA-$FAM46C^{mut}$ (**a, b**; lane 3 and 6) tethering. Expression of NHA-tagged FAM46C proteins were confirmed using an α-HA antibody. DBC1 and RPS5 served as a loading control. Asterisk indicates non-specific signals for HA and FLuc antibodies.

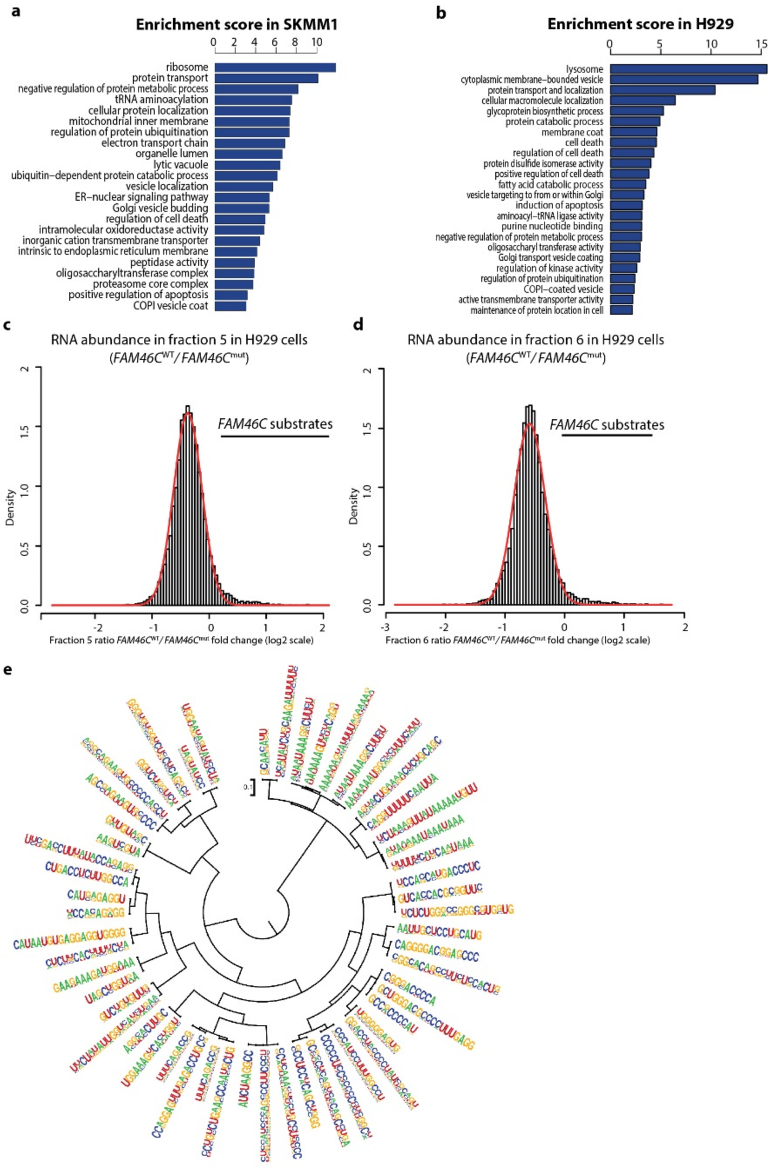

Supplementary Figure 7. Analysis of FAM46C substrates in MM cells. (**a, b**) Functional GO terms annotation clustering of significantly upregulated transcripts in FAM46C^{WT} overexpressing SKMM1 (**a**) and H929 (**b**) cell lines. (**c, d**) Histogram representing the distribution of FAM46C-dependent polyadenylation rations monitored in fraction #5 (**c**) and #6 (**d**) in H929 cell lines. Modeled normal distribution is fitted as a red line to emphasize the outlying population of FAM46C polyadenylated transcripts. (**e**) The sequence composition of significantly overrepresented motifs (P value $<10^{-12}$) in 3'UTRs of FAM46C substrates. The distance was calculated based on the Pearson Correlation Coefficient and an unweighted pair group method with arithmetic mean (UPGMA) tree-construction algorithms was used.

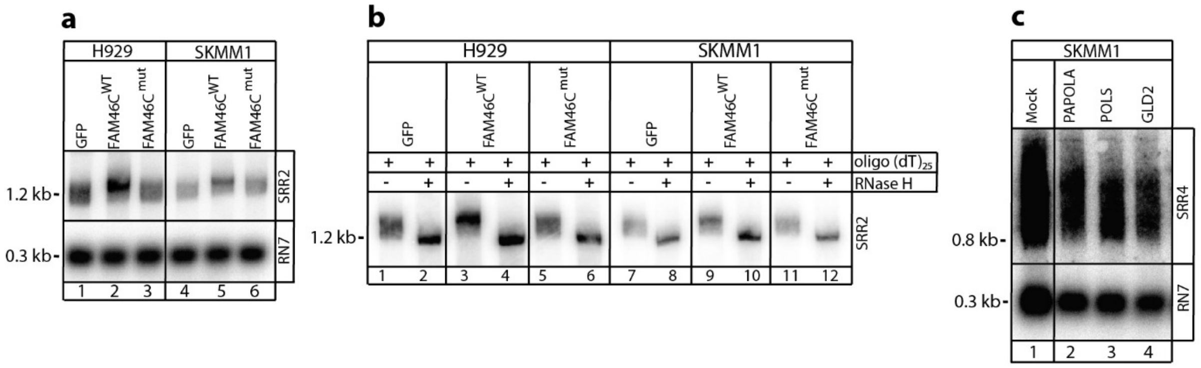

Supplementary Figure 8. *SSR2* mRNA, similarly to *SSR4*, is polyadenylated by FAM46C. (**a)** High resolution northern blot analysis of *SSR2* transcript from MM cell lines H929 (lanes 1-3), SKMM1 (lanes 4-6) transduced with GFP (lanes 1, 4), FAM46C^{WT}-GFP (lanes 2, 5), and FAM46C^{mut}-GFP (lanes 3, 6). High-resolution northern blot analyses were performed using 4% denaturing PAGE gel. (**b**) High-resolution northern blot analysis of *SSR2* transcripts from H929 (lanes 1-6) and SKMM1 cells (lanes 7-12) transduced with GFP (lanes 1, 2, 7, 8), FAM46C^{WT}-GFP (lanes 3, 4, 9, 10), and FAM46C^{mut}-GFP (lanes 5, 6, 11, 12) after RNase H treatment (lanes 2, 4, 6, 8, 10, 12) to remove the poly(A) tail in the presence of oligo(dT)$_{25}$. Control reactions were carried out in the presence of oligo(dT)$_{25}$ without RNase H (lanes 1, 3, 5, 7, 9, 11). (**c**) *SSR4* is not the substrate for PAPOLA, POLS, and GLD2. High-resolution northern blot analysis of *SSR4* transcripts from SKMM1 cells transduced with lentiviruses carrying *PAPOLA* (lane 2), *POLS* (lane 3), *GLD2* (lane 4), and control cells (lane 1). RN7 RNA served as a loading control.

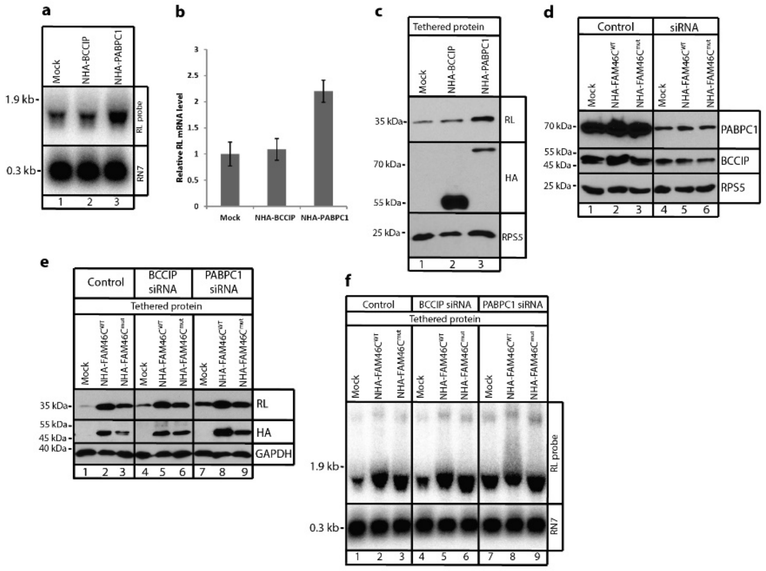

Supplementary Figure 9. (**a-c**) Tethering of PABPC1 to reporter RL mRNA leads to increase of its steady state level. (**a**) Northern blot analysis of total RNA from HEK293 cells transfected with pRL-5BoxB and plasmids encoding NHA-BCCIPβ (lane 2) and NHA-PABPC1 (lane 3) using a probe against *Renilla* luciferase. Cells transfected with pRL-5BoxB only were used as controls (lane 1). Membrane methylene blue staining shown as a loading control. (**b**) Quantification of northern blots shown in panel **a** using Multigauge software. Bars represent mean values ± SD (n=3). (**c**) Western blot detection of NHA-BCCIPβ (lane 2) and NHA-PABPC1 (lane 3) with α-HA antibodies and reporter-encoded *Renilla* luciferase proteins using α-RL antibodies in control cells (lane 1). (**d-f**) Silencing of FAM46C interactors has no effect on its activity in tethering assays. (**d**) Western blot detection of BCCIP or PABPC1 proteins in control cells (lanes 1-3) and after siRNA-mediated knockdowns (lanes 4-6). FAM46C tethering cells were transfected with pRL-5BoxB only (lanes 1, 4) or co-transfected additionally with plasmids encoding NHA-FAM46C^{WT} (lanes 2, 5), and NHA-FAM46C^{mut} (lanes 3, 6). (**e**) Western blot detection of NHA-FAM46C^{WT} and NHA-FAM46C^{mut} proteins using α-HA antibodies, *Renilla* luciferase proteins with an α-RL antibody in control HEK293 cells (lanes 1-3), or reduced levels of BCCIP (lanes 4-6) or PABPC1 (lanes 7-9). (**f**) Northern blot analysis of total RNA from control HEK293 cells (lanes 1-3) or with reduced levels of BCCIP (lanes 4-6) or PABPC1 (lanes 7-9) transfected with pRL-5BoxB only (lanes 1, 4, 7) or co-transfected additionally with plasmids encoding NHA-FAM46C^{WT} (lanes 2, 5, 8) or NHA-FAM46C^{mut} (lanes 3, 6, 9) using probes against *Renilla* luciferase. Membrane methylene blue staining is shown as a loading control.

a

Figure 1b

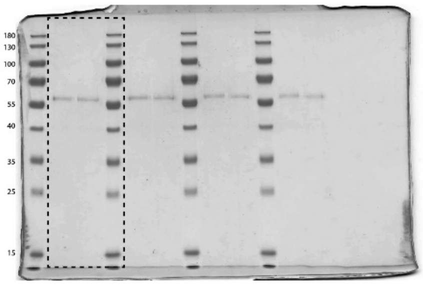

b

Figure 1d

Autoradiography

Ponceau S Staining

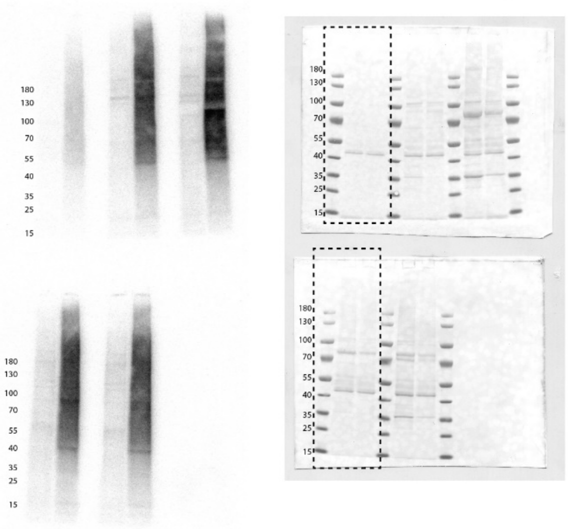

c

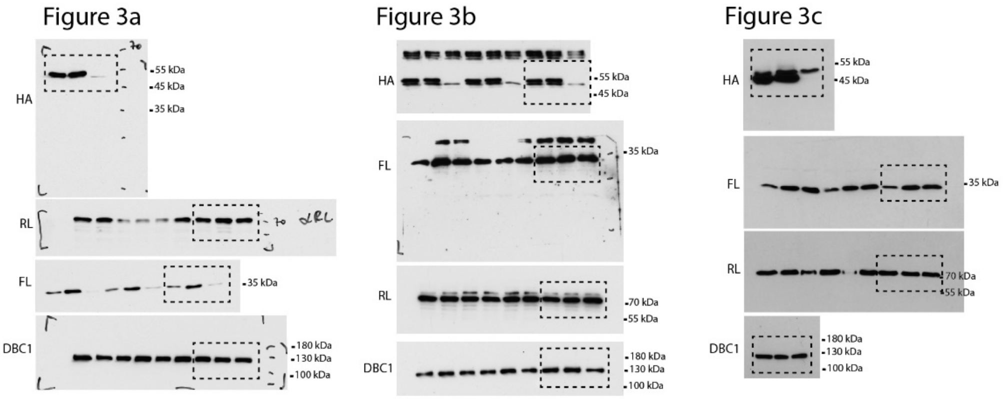

Figure 3a
Figure 3b
Figure 3c
HA
RL
FL
DBC1
55 kDa
45 kDa
35 kDa
70 kDa
180 kDa
130 kDa
100 kDa

d

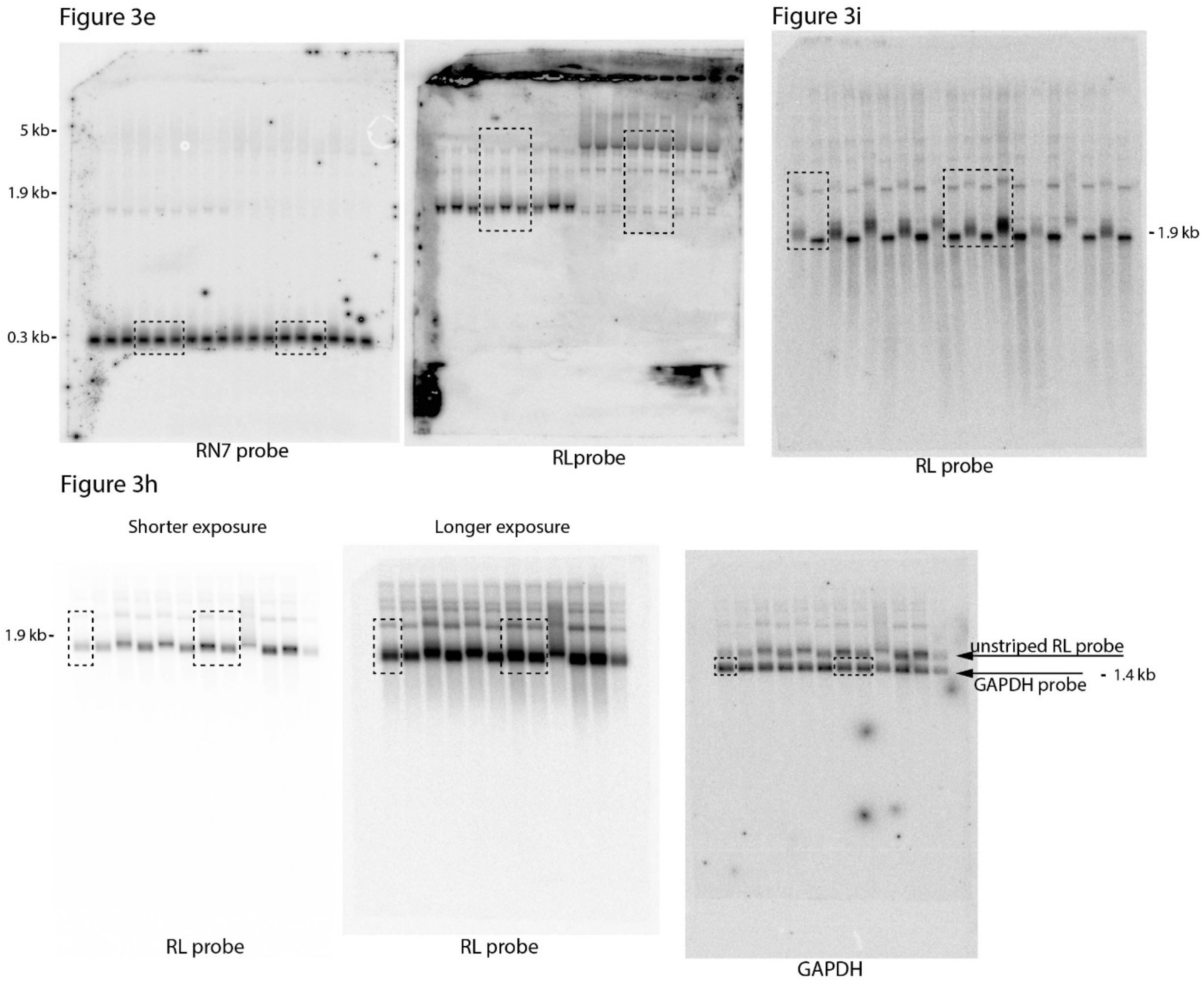

Figure 3e
Figure 3i
5 kb
1.9 kb
0.3 kb
RN7 probe
RLprobe
RL probe
Figure 3h
Shorter exposure
Longer exposure
RL probe
RL probe
GAPDH
unstriped RL probe
GAPDH probe
1.4 kb

e

Figure 5a

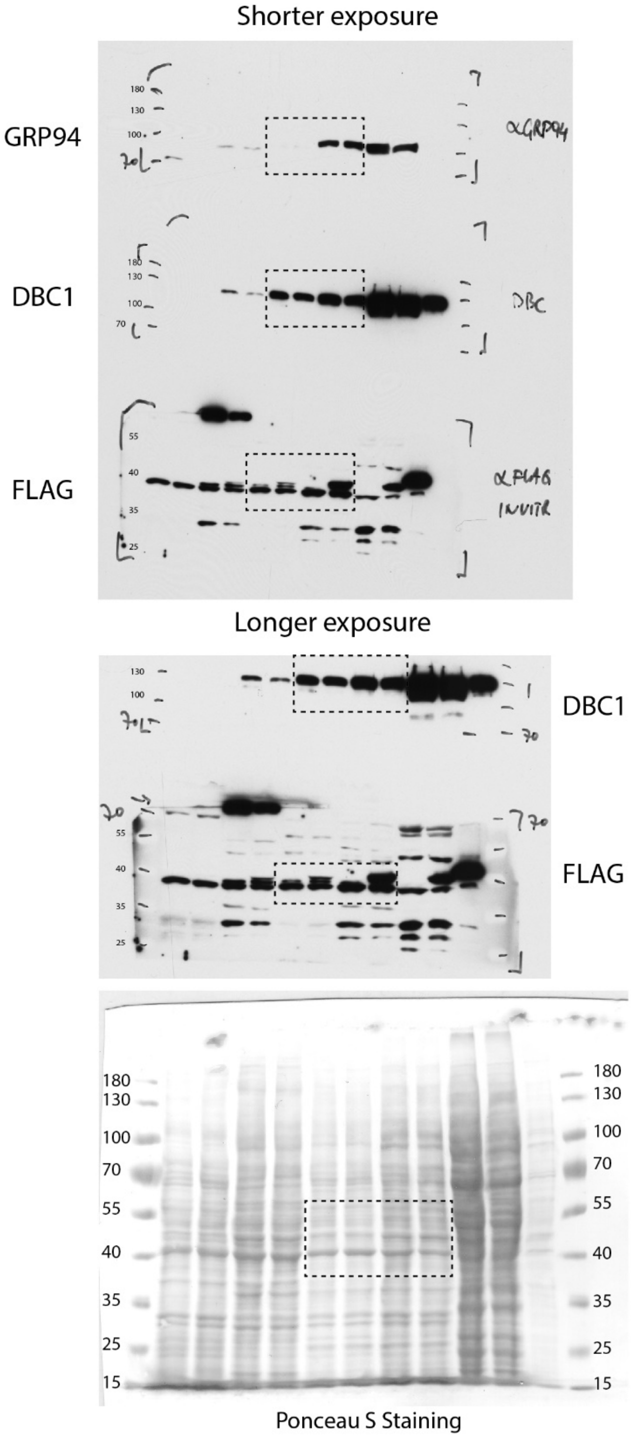

Figure 5b

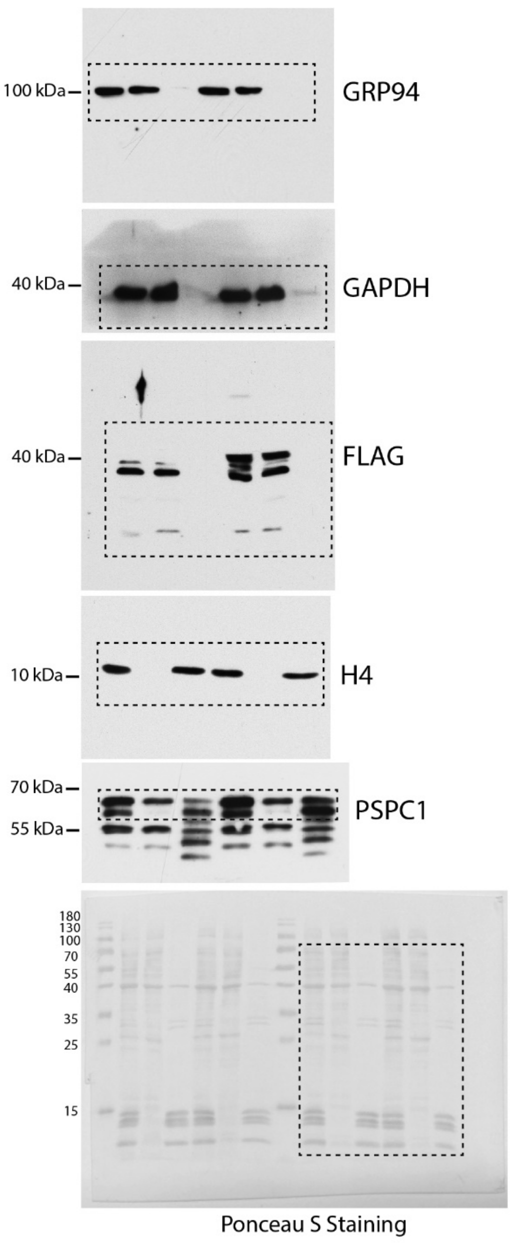

f

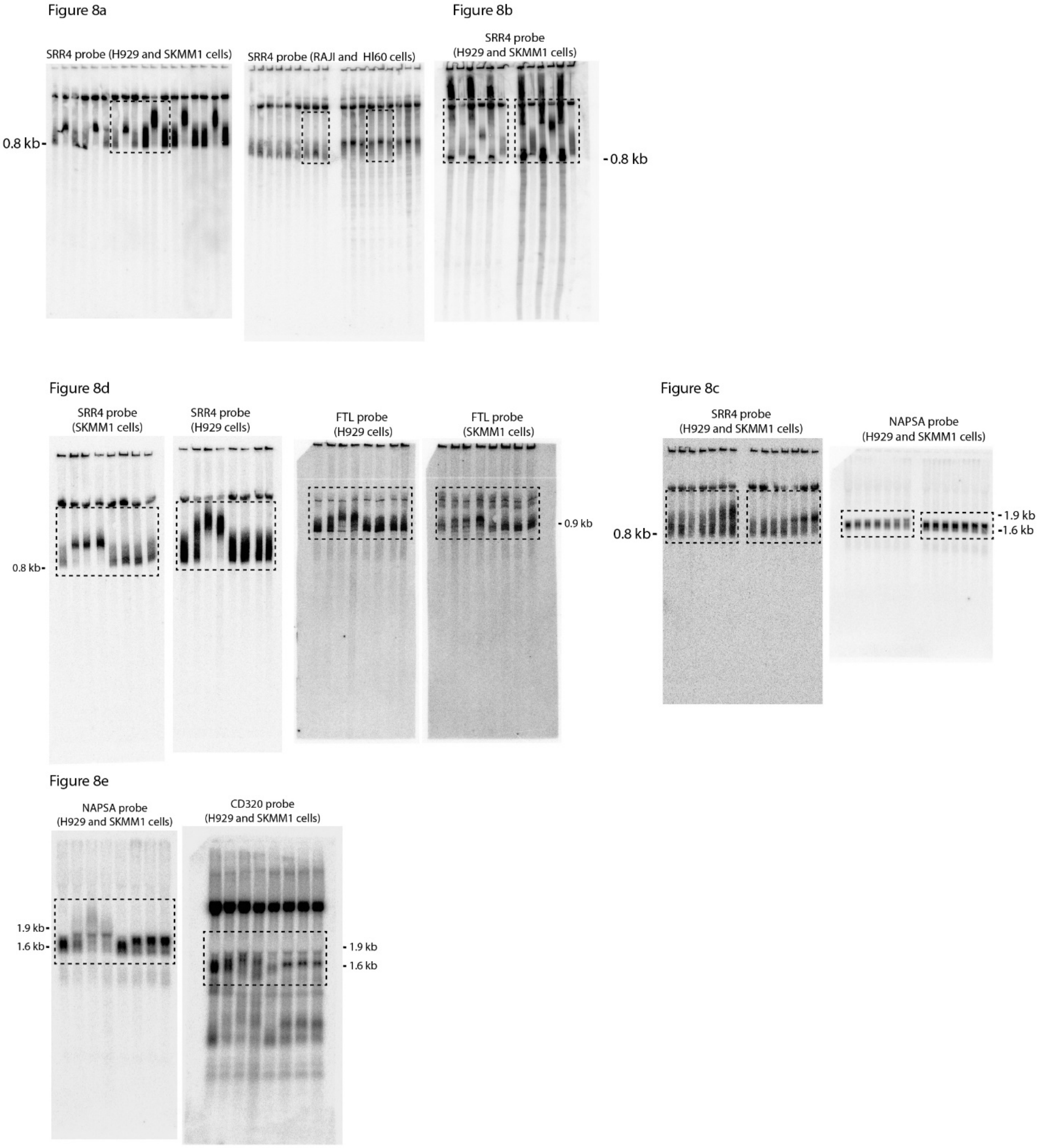
Figure 8a
SRR4 probe (H929 and SKMM1 cells)
SRR4 probe (RAJI and Hl60 cells)
0.8 kb
Figure 8b
SRR4 probe
(H929 and SKMM1 cells)
0.8 kb
Figure 8d
SRR4 probe
(SKMM1 cells)
SRR4 probe
(H929 cells)
FTL probe
(H929 cells)
FTL probe
(SKMM1 cells)
0.8 kb
0.9 kb
Figure 8c
SRR4 probe
(H929 and SKMM1 cells)
NAPSA probe
(H929 and SKMM1 cells)
0.8 kb
1.9 kb
1.6 kb
Figure 8e
NAPSA probe
(H929 and SKMM1 cells)
CD320 probe
(H929 and SKMM1 cells)
1.9 kb
1.6 kb
1.9 kb
1.6 kb

g

Figure 9b

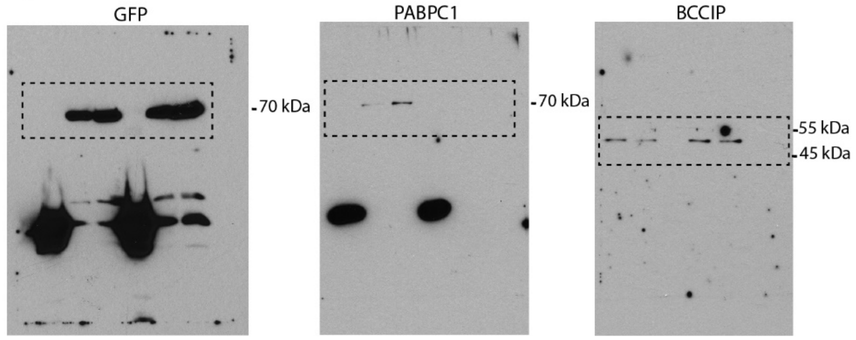

Figure 9b

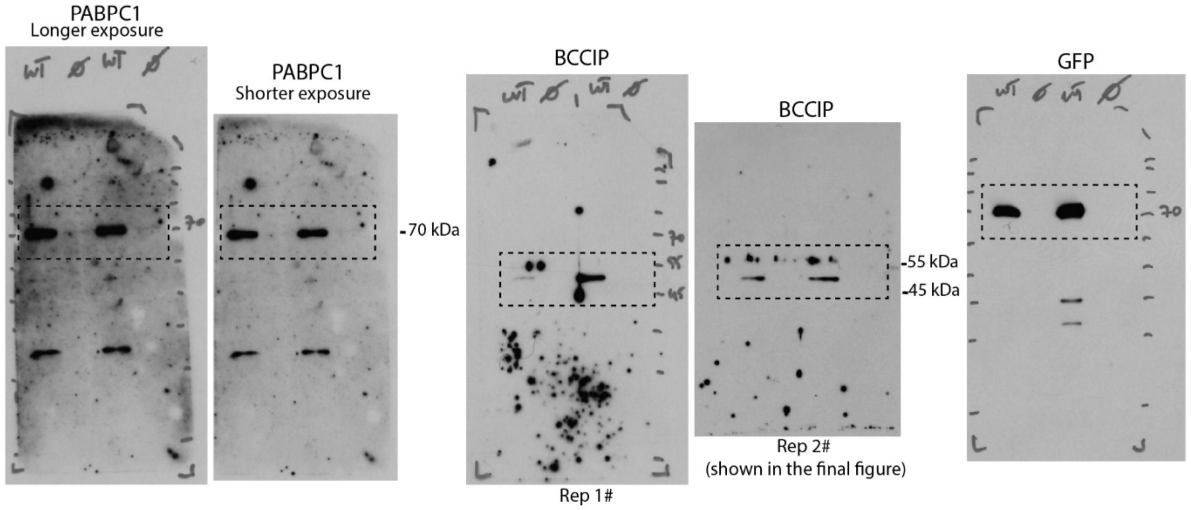

Supplementary Figure 10. Unprocessed scans of selected blots and gels. **(a)** Scan of gel corresponding to Fig. 1b. **(b)** Blots corresponding to Fig. 1d. **(c)** Western blots corresponding to Fig. 3a, Fig. 3b, Fig. 3c. **(d)** Northern blots corresponding to Fig. 3e, 3h, 3i. **(e)** Western blots and membrane Ponceau S staining corresponding to Fig. 5a and Fig. 5b. **(f)** Northern blots corresponding to all panels of Fig. 8. **(g)** Western blots corresponding to Fig. 9b and Fig. 9c.

Supplementary Table 1. Cell lines used in this study.

**Multiple myeloma cell line**	**Mutations in *FAM46C* gene**
SKMM1	Homozygotic deletion c.519delT (p.I173fsX36)
H929	Hemizygotic insertion c.278-279insC (p.L93fsX15)
MM1.S	Hemizygotic substitution c.808A>G (p.M270V)
RPMI8226	None

Supplementary Table 2. Antibodies used in this study.

**Antibodies and Cell Isolation Kits**	**Manufacturer and catalog number**
Anti-*Renilla* Luciferase	Millipore; Anti-Renilla Luciferase clone 5B11.2; MAB4400
Anti-*Firefly* Luciferase	Abcam; Anti-Firefly Luciferase antibody; ab21176
Anti-BCCIP	Abcam; Anti-BCCIP antibody; ab97577
Anti-HA	Abcam; Anti-HA tag antibody [HA.C5]; ab18181
Anti-PABPC1	Abcam; Anti-PABP antibody; ab21060
Anti-DBC1	Bethyl Lab; DBC1/p30 DBC Antibody; A300-434A
Anti-FLAG	Invitrogen; DYKDDDDK Tag Polyclonal Antibody; PA1-984B
Anti-GFP	Santa Cruz; GFP Antibody (B-2); sc-9996
Anti-RPS5	Santa Cruz; Ribosomal Protein S5 (464-J); sc-100832
Anti-GAPDH	Novus Biologicals; anti-GAPDH antibody; NB300-327
Anti-SSR4	Abcam; Anti-Signal sequence receptor delta; ab58009
Anti-Histone H4	Anti-Histone H4, pan, clone 62-141-13; 05-858
Anti-FAM46C 1	Abcam; Anti-FAM46C antibody; ab74754
Anti-FAM46C 2	Abcam; Anti-FAM46C antibody; ab169699
Anti-FAM46C 3	Santa Cruz; FAM46C (D-14); sc-164330
Anti-FAM46C 4	Santa Cruz; FAM46C (P-12); sc-164332
Anti-GRP94	Santa Cruz; GRP 94 (9G10); sc-32249

anti-CD20-Alexa Fluor 700	Novus Biologicals; MS4A1/CD20 Antibody (AISB12); NBP1-43435AF700
Anti-CD138-PE	DB; BD 553714
anti-CD45.2 Horizon V500	DB; anti-Mouse CD45.2 (Clone 104); BD 562129
Anti-PSPC1	Abcam; Anti-PSPC1 antibody (ab104238)
B Cell Isolation Kit, mouse	Miltenyi Biotec; 130-090-862
CD138+ Plasma Cell Isolation Kit, mouse	Miltenyi Biotec; 130-092-530

Supplementary Table 3. siRNAs used in this study.

**Targeted gene**	**Stealth Select RNAi™ siRNA reference number**	**Name used in this study**
BCCIP	HSS125584	siBCCIP_1
BCCIP	HSS125585	siBCCIP_2
BCCIP	HSS125586	siBCCIP_3
PABPC1	HSS167099	siPABPC1_1
PABPC1	HSS167100	siPABPC1_2
PABPC1	HSS167101	siPABPC1_3

Supplementary Table 4. shRNAs used in this study.

**construct**	**Catalog or TRC Number**	**Sequence of insert**
pLKO.1-Empty	SHC001 (pLKO.1-puro Empty Vector)	No shRNA Insert
sh2 (hFAM46C –sh2)	TRCN0000168752	CCGGGCAGAATTTCAGCTGGTTAGACTCGAGTCTAACCAGCTGAAATTCTGCTTTTTG
sh3 (hFAM46C –sh3)	TRCN0000166958	CCGGGCCTAAATCTTGTTTACCTATCTCGAGATAGGTAAACAAGATTTAGGCTTTTTG
sh5 (hFAM46C –sh5)	TRCN0000172684	CCGGGCTGAAGTTTGTCGACTCCATCTCGAGATGGAGTCGACAAACTTCAGCTTTTTG

Supplementary Table 5. Genotypes of mice used in this study.

**Mouse lines**	**Mutations in *FAM46C* gene**
FAM46C-/- mouse line	Homozygotic indel c.261_271delins301nt[1] (p.G85fsX26)
FAM46C-FLAG mouse line	Homozygotic insertion c.1176ins24bp[2] (p.N391insDYKDDDDK)

[1] – apparently this indel consists of c.261_271delTTGCAAAGATCTGGATC (g.16015_16061del) and insertion of translocated intronic duplication g.15373_15670 fragment:

c.261_271delTTGCAAAGATCTGGATCinsAACCGACAGGGTCGATTTCTCTCTGCGGT
TAGAGGTCCTTCATTGTTTAGATGGCATGGTTAGTTTGCTATTCTTCATCTAAAGC
AGTCCCCCTTTCCTCTCCCCCACATAGGTCAAGCACACCAGCTAGAGCACCCACA
CAAAGCAAAGTAGTGCATTACCACCCTGCTGTAGAACGGAGCTTGTGCCTGGCTG
GGTATTACTCTTGGTCGCAGCCGTGTGGAGCAGCAGGCACTGCTGACTGCTGTTT
TGCAGGAAATTGTTCAGAGATGGGTCAGTGCCTCTGAACTGACTCGTCCGT

[2] – full sequence of the c.1176ins24bp: insGACTACAAAGACGATGACGACAAG
