# Supplementary material for: The non-canonical poly(A) polymerase FAM46C acts as an onco-suppressor in multiple myeloma
Source: Nat Commun. 2017 Sep 20;8:619. doi: 10.1038/s41467-017-00578-5 (PMC5606997; doi:10.1038/s41467-017-00578-5)
Supplement: Supplementary file 6 — Supplementary Data 4 [file 41467_2017_578_MOESM6_ESM.docx]

**Supplementary Data 4. List of oligonucleotides used in this study.**

| **Oligonucleotide** | **Sequence** | | **Purpose** |
| --- | --- | --- | --- |
| NHABCCIPF (*EcoRI* site) | ACGCTGAATTCATGGCGTCCAGGTCTAAGCGGCGTG | | Cloning into pClneo-NHA |
| NHABCCIPR (*NotI* site) | GGGAAGCGGCCGCTTAGACAGATAGATATTCTTTCAG | | Cloning into pClneo-NHA |
| NHAPABPC1F  (*SalI* site) | CTAGAGTCGACATGAACCCCAGTGCCCCCAGCTAC | | Cloning into pClneo-NHA |
| NHAPABPC1R  (*NotI* site) | GGGAAGCGGCCGCTTAAACAGTTGGAACACCGGTGGC | | Cloning into pClneo-NHA |
| NHFAM46CF | GATTACGCTTCACTCGACGCGGATCCCGTCGAATTC ATGGCAGAGGAGAGCAGCTGTACCAGG | | Cloning into pClneo-NHA |
| NHFAM46CR | GCTCGAAGCATTAACCCTCACTAAAGGGAAGCGGCCGCCTAGTTACAGGGCAGCCAGGTAGGGTAAGG | | Cloning into pClneo-NHA |
| NHFAM46DF | GATTACGCTTCACTCGACGCGGATCCCGTC GAATTCatgtctgaaatcagattcaccaatct cac | | Cloning into pClneo-NHA |
| NHFAM46DR | GCTCGAAGCATTAACCCTCACTAAAGGGAAGCGGCCGCttaactcataccatttgatccacgaaagtg | | Cloning into pClneo-NHA |
| pcDNAFam46CF | GGATCCGAAAACCTGTACTTCCAAGGAACCGGTATGGCAGAGGAGAGCAGCTG | | pcDNA-GFP construct |
| pcDNAFam46CR | GATATCACCCTGAAAATACAAATTCTCGCTAGCGTTACAGGGCAGCCAGGTAG | | pcDNA-CGFP construct |
| pcDNABCCIPF | GGATCCGAAAACCTGTACTTCCAAGGAACCGGTatggcgtccaggtctaagcggc | | pcDNA-CGFP construct |
| pcDNABCCIPR | GATATCACCCTGAAAATACAAATTCTCGCTAGCgacagatagatattctttcag | | pcDNA-CGFP construct |
| pcDNAPABPC1F | GGATCCGAAAACCTGTACTTCCAAGGAACCGGTatgaaccccagtgcccccag | | pcDNA-CGFP construct |
| pcDNAPABPC1R | GATATCACCCTGAAAATACAAATTCTCGCTAGCaacagttggaacaccggtgg | | pcDNA-CGFP construct |
| pcDNAGld2F | GGATCCGAAAACCTGTACTTCCAAGGAACCGGTATGTTCCCAAACTCAATTTTG | | pcDNA-CGFP construct |
| pcDNAGld2R | GATATCACCCTGAAAATACAAATTCTCGCTAGCTCTTTTCAGGACAGCAGCTC | | pcDNA-CGFP construct |
| pcDNAPOLSF | GGATCCGAAAACCTGTACTTCCAAGGAACCGGTatgtccccttgtcctgaagaag | | pcDNA-CGFP construct |
| pcDNAPOLSR | GATATCACCCTGAAAATACAAATTCTCGCTAGCtctgctgaggctcacgggcag | | pcDNA-CGFP construct |
| pcDNAPAPOLAF | GGATCCGAAAACCTGTACTTCCAAGGAACCGGTatgccgtttccagttacaacacagg | | pcDNA-CGFP construct |
| pcDNAPAPOLAR | GATATCACCCTGAAAATACAAATTCTCGCTAGCccgattcaatctcagttttattg | | pcDNA-CGFP construct |
| pcDNAFAM46CmutF | GGGCTGCAAAGCCCTGGCCCTAATCTTCC | | FAM46CWT mutagenesis |
| pcDNAFAM46CmutF | GGAAGATTAGGGCCAGGGCTTTGCAGCCC | | FAM46CWT mutagenesis |
| pcDNAFAM46DmutF | agctataagGCCctgGCCgttatttttg | | FAM46DWT mutagenesis |
| pcDNAFAM46DmutR | caaaaataacGGCcagGGCcttatagct | | FAM46DWT mutagenesis |
| Fam46D_NHISF | TGAAGTCTACCAGGAACAAACCGGTGGATCCATGTCTGAAATCAGATTCAC | | Cloning into pET28 vector (N-terminal HisTAG-Sumo) |
| Fam46D_NHISR | GATCTCAGTGGTGGTGGTGGTGGTGCTCGAGTTAACTCATACCATTTGATC | | Cloning into pET28 vector  N-terminal HisTAG-Sumo) |
| Fam46C_NHISF | TGAAGTCTACCAGGAACAAACCGGTGGATCCATGGCAGAGGAGAGCAGCTG | | Cloning into pET28 vector  N-terminal HisTAG-Sumo) |
| Fam46C_NHISF | GATCTCAGTGGTGGTGGTGGTGGTGCTCGAGTTAGTTACAGGGCAGCCAGG | | Cloning into pET28 vector  (N-terminal HisTAG-Sumo) |
| GAPDHR | CATACCAGGAAATGAGCTTG | | GAPDH Northern blot probe |
| GAPDHF | GGATATTGTTGCCATCAATG | | GAPDH Northern blot probe |
| RLucF | atgattactggtccacaatgg | | RL Northern blot probe |
| RLucR | gaacacgctcaacaaacgat | | RL Northern blot probe |
| FTL1 | GAGAGGTAGGTGTAGGAGGC | | FTL1 probe |
| Cd320F | ATGAGCGGCGGTTGGATGGC | | CD320 Northern blot probe |
| Cd320R | CGTCGCTGGAGTCGGGACAG | | CD320 Northern blot probe |
| NAPSAF | CTCTTCATCGAGTCCAACCTG | | NAPSA Northern blot probe |
| NAPSAR | AGACAGGCTTATCCAATAGC | | NAPSA Northern blot probe |
| SSR4F | ATGGCGGCGATGGCATCTCTCG | | SSR4 Northern blot probe |
| SSR4R | GGCCTGGATGTGGCTCTTCGC | | SSR4 Northern blot probe |
| SSR2F | TTGCTGTCACTCAAGCAGAGG | | SSR2 Northern blot probe |
| SSR2R | TCCTCTTGCTGGAGTACCAC | | SSR2 Northern blot probe |
| RN7 (RN7SL1) | CACTACTGATCAGCACGGGAG | | 7SL Northern blot probe |
| hFam46C_ex2_1F | CTACTGCGGTCCTATATGGTT | | FAM46C amplification for sequencing |
| hFam46C_ex2_1R | GCCCAAATTCTCCCAATCTCG | | FAM46C amplification for sequencing |
| Fam46C_ex2_2F | ACACTGACCGCTGGAGCCTG | | Internal sequencing primers FAM46C |
| Fam46C_ex2_2R | CCTGAAGTCCCGCACAAGA | | Internal sequencing primers FAM46C |
| SFFV_R | CATCGATAAGCTTGATATCAAGCTTGCATGCTCGAGCTATCTAGATCCGGTGGATCCCGG | | Universal primer for cloning into  HIVSFFV |
| SFFV_F | GTCCTCCGACAGACTGAGTCGCCCGGGGGGGAAAACCTGTACTTCCAAGGAACC | | Universal primer for cloning into HIVSFFV |
| FL1F | GTGCCAGAGTCCTTCGATAG | | qPCR (Firefly luciferase). |
| FL1R | CTCACGCAGGCAGTTCTATG | | qPCR (Firefly luciferase). |
| FL2F | GTTGTTCCATTCCATCACGG | | qPCR (Firefly luciferase). |
| FL2R | CCTGAAGGCTCCTCAGAAAC | | qPCR (Firefly luciferase). |
| FAM46CF1 | TCTTCATCGACTTCCCGGACA | | qPCR (FAM46C) |
| FAM46CR1 | GACACACGGTGCTCTCGTTC | | qPCR (FAM46C) |
| FAM46CF2 | ACTTCCTGCCAGAGGGTGTG | | qPCR (FAM46C) |
| FAM46CR2 | AGGCTCCAGCGGTCAGTGTC | | qPCR (FAM46C) |
| GAPDHF | AAGGTGAAGGTCGGAGTCAAC | | qPCR |
| GAPDHR | GGGGTCATTGATGGCAACAATA | | qPCR |
| 15_131nF | CGACAGACTGAGTCGCCCGGGGGGGGATCCATGACTTCGAAAGTTTATG | | Cloning of RL and RL5Box into  HIVSFFV |
| 15_131R1 | GTAGTTGGACTTATCTAGATTATTGTTCATTTTTGAGAAC | | Cloning of RL into  HIVSFFV |
| 15_131F2 | GTTCTCAAAAATGAACAATAATCTAGATAAGTCCAACTAC | | Cloning of RL5Box into  HIVSFFV |
| 15_131_R | TAAGCTTGATATCAAGCTTGCATGctcgagAAAGGGAAGCGGCCGCCTCG | | Cloning of RL5Box into  HIVSFFV |
| 15_133_F | CGACAGACTGAGTCGCCCGGGGGGGGATCCccatggaagacgccaaaaac | | Cloning of FL into  HIVSFFV |
| 15_133_R | TAAGCTTGATATCAAGCTTGCATGCTCGAGttacacggcgatctttccgc | | Cloning of FL into  HIVSFFV |
| HIV_f | GAGCTCTAAAAAGAGCTCAC | | HIVSFFV constructs sequencing |
| HIV_r | GTTAAGAATACCAGTCAATC | | HIVSFFV constructs sequencing |
| EGFP_F | catggtcctgctggagttcg | | sequencing of all constructs with N-terminal GFP tag |
| EGFP_R | tgaacttgtggccgtttacg | | sequencing of all constructs with C-terminal GFP tag |
| FRTTO_F | tgacctccatagaagacacc | | sequencing of pKK (pcDNA5FRTTO) insert |
| FRTTO_R | aactagaaggcacagtcgag | | sequencing of pKK (pcDNA5FRTTO) insert |
| N_Met-HA_for | ATGTACCCCTACGACGTGCCCGACTACGCTGGATCCGAAAACCTGTACTTCCA | | Insertion of HA into pCDNA |
| N_Met-HA_rev | AGCGTAGTCGGGCACGTCGTAGGGGTACATGGTACCAAGCTTAAGTTTAAACG | | Insertion of HA into pCDNA |
| 15_129_F | CGACAGACTGAGTCGCCCGGGGGGGGATCCATGGACGCACAAACACGACG | | Cloning of NHAFAM46C into HIVSFFV |
| 15_129_R | TGATATCAAGCTTGCATGCTCGAGGTCGACttagttacagggcagccagg | | Cloning of NHAFAM46C into HIVSFFV |
| 15_125F1 | Catggattacaaggatgacgacgataagggatccgaaaacctgtacttcc | | Cloning of FLAGFAM46C into HIVSFFV |
| HivNFlgF | GTCCTCCGACAGACTGAGTCGCCCGGGGGGGGATCCatggattacaaggatgacg | | Cloning of FLAGFAM46C into HIVSFFV |
| HivN_R | CATCGATAAGCTTGATATCAAGCTTGCATGCTCGAGatcaccctgaaaatacaaattctc | | Cloning of FLAGFAM46C into HIVSFFV |
| HIV_for | GTCCTCCGACAGACTGAGTCGCCCGGGGGGGAAAACCTGTACTTCCAAGGAACC | | Cloning of FAM46CFLAG into HIVSFFV |
| 15_127R1 | CATGCTCGAGctacttatcgtcgtcatccttgtaatcgatatcaccctgaaaatac | | Cloning of FAM46CFLAG into HIVSFFV |
| HivCflgR | CATCGATAAGCTTGATATCAAGCTTGCATGCTCGAGctacttatcgtcgtcatcc | | Cloning of FAM46CFLAG into HIVSFFV |
| HivNgfpF | GTCCTCCGACAGACTGAGTCGCCCGGGGGGGGATCCatggtgagcaagggcgagg | | Cloning of GFP-FAM46C into HIVSFFV |
| 15_123R1 | ggttccttggaagtacaggttttcggatcccttgtacagctcgtccatgc | | Cloning of GFP-FAM46C into HIVSFFV |
| 9_114_F | GGATCCGAAAACCTGTACTTCCAAGGAACCGGTATGGCAGAGGAGAGCAGCTG | | Cloning of GFP-FAM46C into HIVSFFV |
| FAM46C knock-out guide RNA | | CGGCTTGGGTTGCAAAGATC**TGG** | guide RNA for CRISPR-based generation of FAM46C catalytic/knock-out mutations in mice |
| FAM46C FLAG knock-in guide RNA | | CAGGTCTTCAGGTTAGTTAC**AGG** | guide RNA for CRISPR-based generation C-terminal FLAG tagged FAM46C in mice |
| mFam46C_mut_sgRNA_f | | TAATACGACTCACTATAGGG**CGGCTTGGGTTGCAAAGA**TCGTTTTAGAGCTAGAAATAGCAAGTTAAAATAAGGC | Forward primer (inc. T7 Pol RNA promoter) for generation of sgRNA |
| mFam46C_FLAG_sgRNA_f | | gaaattaatacgactcactatagggAGGTCTTCAGGTTAGTTACgttttagagctagaaatagcaagttaaaataaggc | Forward primer (inc. T7 Pol RNA promoter) for generation of sgRNA |
| Universal_sgRNA_rev | | AAAAAGCACCGACTCGGTGCCACTTTTTCAAGTTGATAACGGACTAgccttattttaacttgctatttctagctcta | Reverse primer for construction of sgRNAs |
| mFam46C_FLAG_oligo | | CCTCCAATTACCTACAGCCAGCCTTATCCTACATGGCTGCCCTGTAACGACTACAAAGACGATGACGACAAGTAAcctgaagacctgagggtttccacagtgggaactcggttagggcag | ODN donor for CRISPR-based generation of C-terminal FLAG tagged knock-in FAM46C mice |
| mF46C_seq2F | | AGGTCCTGACTGAGGTCGTG | Forward sequencing primer for mice FAM46C mutations genotyping |
| mF46C_seq2R | | TTCCTCAAAATCCCCGTACA | Reverse sequencing primer for mice FAM46C mutations |
| mF46cFLAG_seq1F | | CTTCAGAACCACTTCTCGGA | Forward sequencing primer of FAM46C--FLAG-tagged mice genotyping |
| mF46cFLAG_seq1R | | CTTCAGAACCACTTCTCGGA | Reverse sequencing primer of FAM46C- FLAG-tagged mice |
| T7_SpCas9_for | | TGTAATACGACTCACTATAGGGAGAATGGACTATAAGGACCACGAC | Substrate for T7-based Cas9 mRNA synthesis. Forward primer |
| SpCas9_rev | | CCTCTCCACTGCCgaattaC | Substrate for T7-based Cas9 mRNA synthesis. Reverse primer |
